# On the computational modeling of the innate immune system

**DOI:** 10.1186/1471-2105-14-S6-S7

**Published:** 2013-04-17

**Authors:** Alexandre Bittencourt Pigozzo, Gilson Costa Macedo, Rodrigo Weber dos Santos, Marcelo Lobosco

**Affiliations:** 1 Graduate Program in Computational Modeling, UFJF, Rua José Lourenço Kelmer, s/n -Campus Universitário, Bairro São Pedro, CEP: 36036-900, Juiz de Fora, MG, Brazil; 2 Graduate Program in Biological Sciences, UFJF, Rua José Lourenço Kelmer, s/n - Campus Universitário, Bairro São Pedro, CEP: 36036-900, Juiz de Fora, MG, Brazil

## Abstract

In recent years, there has been an increasing interest in the mathematical and computational modeling of the human immune system (HIS). Computational models of HIS dynamics may contribute to a better understanding of the relationship between complex phenomena and immune response; in addition, computational models will support the development of new drugs and therapies for different diseases. However, modeling the HIS is an extremely difficult task that demands a huge amount of work to be performed by multidisciplinary teams. In this study, our objective is to model the spatio-temporal dynamics of representative cells and molecules of the HIS during an immune response after the injection of lipopolysaccharide (LPS) into a section of tissue. LPS constitutes the cellular wall of Gram-negative bacteria, and it is a highly immunogenic molecule, which means that it has a remarkable capacity to elicit strong immune responses. We present a descriptive, mechanistic and deterministic model that is based on partial differential equations (PDE). Therefore, this model enables the understanding of how the different complex phenomena interact with structures and elements during an immune response. In addition, the model's parameters reflect physiological features of the system, which makes the model appropriate for general use.

## Introduction

The human immune system (HIS) consists of a wide and complex network of cells, tissues and organs. The HIS plays a crucial role in defending the body against disease. To achieve this goal, the HIS identifies and kills a wide range of external pathogens such as viruses and bacteria as well as the body's own abnormally behaving cells. The HIS is also responsible for removing dead cells and regenerating some of the body's structures [1].

A complete understanding of the HIS is therefore essential. However, its complexity and the intense interactions among several components on various different levels make this task extremely complex [2,3]. However, we may better understand some properties of the HIS by applying a computational model, which allows researchers to test a large number of hypotheses in a short period of time [2,3]. In the future, we can envision a computer program that will simulate the entire HIS, allowing scientists to develop and test new drugs against various diseases virtually, thus reducing the number of animals used in experiments.

In this study, our work aims to implement and simulate a mathematical model of the HIS. Due to the complexity of this task, our focus is to reproduce the spatio-temporal dynamics of an immune response to the injection of lipopolysaccharides (LPS) into a small section of tissue. To reproduce these dynamics, we introduce a mathematical model composed of a system of partial differential equations (PDEs) that extends our previous model [2] and defines the dynamics of representative cells and molecules of the HIS during the immune response to LPS. The model presented is descriptive, mechanistic and deterministic; therefore, it enables the understanding of how different complex phenomena, structures and elements interact during an immune response. In addition, the model's parameters reflect the physiological features of the system, making the model appropriate for general use.

The remainder of the paper is organized as follows. First, the necessary biological background is presented. Next, related works are briefly discussed. This exposition is followed by a description of both the mathematical model proposed in this work and its computational implementation. Then simulation results obtained from the proposed model are discussed, and finally, our conclusions and plans for future work are presented.

## Biological background

"Human body surfaces are protected by epithelia, which provide a physical barrier between internal and external environments. Epithelia make up the skin and lining of the tubular structures of the body (i.e., the gastrointestinal, respiratory and genitourinary tracts), and they form an effective barrier against the external environment. At the same time, epithelia can be crossed or settled by pathogens, causing infections. After crossing the epithelium, the pathogens encounter cells and molecules of the innate immune system, which immediately develop a response" [4].

The body's initial response to an acute biological stress, such as a bacterial infection, is an acute inflammatory response [4]. The strategy of the HIS is to keep some resident macrophages on guard in tissues to look for any signal of infection. When they find such a signal, the macrophages alert neutrophils (also known as polymorphonuclear neutrophils (PMNs)) that their help is required. Because of this communication, the cooperation between macrophages and neutrophils is essential to mount an effective defense against disease. Without macrophages to herd neutrophils toward the location of infection, the latter would circulate indefinitely in the blood vessels, impairing the control of systemic infections [1].

The inflammation of an infectious tissue has many benefits for the control of the infection. In addition to recruiting cells and molecules of innate immunity from blood vessels to the location of the infected tissue, inflammation increases the lymph flux, which contains microorganisms and cells that carry antigens to neighboring lymphoid tissues; there, these cells will present the antigens to the lymphocytes and initiate the adaptive response. Once the adaptive response has been activated, the inflammation also shuttles the effector cells of the adaptive immune system to the location of infection [4].

A component of the cellular wall of Gram-negative bacteria, such as LPS, can trigger an inflammatory response through the interaction with receptors on the surface of some cells [1]. For example, the macrophages that reside in tissue recognize a bacterium through the binding of TLR4 (Toll-like receptor 4) with LPS. When receptors on the surface of macrophages bind to LPS, the macrophage starts to phagocytose, internally weakening the bacterium and secreting proteins known as cytokines and chemokines, as well as other molecules.

In many inflammatory conditions, neutrophils dominate the initial influx of leukocytes into the inflamed tissue. The first wave of extravasated neutrophils is soon replaced by a second wave of monocytes [1]. A study presented initial proofs of the existence of this sequence of events [5]. In that study, neutrophils dominated the leukocyte extravasation three hours after the beginning of the inflammation, and some time later, the extravasated cells were predominantly monocytes [5].

The resolution of the inflammatory response is a complex process that includes the production of anti-inflammatory mediators and the apoptosis (or programmed death) of effector cells of the HIS, such as neutrophils [6]. Anti-inflammatory cytokines form a set of immunoregulatory molecules that control the inflammatory response. These cytokines work together with specific inhibitors and cytokines' soluble receptors to regulate the immune response [6]. A previous work [6] demonstrated the participation of cytokines in inflammatory states. Primary anti-inflammatory cytokines include the antagonist receptor of IL-1 (Interleukin 1) in addition to IL-4, IL-6, IL-10, IL-11 and IL-13 [6]. Specifically, IL-10 is a strong inhibitor of many pro-inflammatory cytokines [7], including IL-8 and TNF-*α *(tumor necrosis factor *α*), which are produced both by monocytes [8] and by neutrophils [9,10].

Apoptotic cells maintain membrane integrity for a small period of time and therefore need to be quickly removed to prevent a secondary necrosis and the consequent release of cytotoxic molecules, which cause inflammation and tissue damage [11]. As a consequence of the phagocytosis of apoptotic cells by macrophages or dendritic cells, these phagocytic cells produce anti-inflammatory cytokines. For example, macrophages secrete TGF-*β *(transforming growth factor *β*), which prevents the release of pro-inflammatory cytokines induced by LPS [12]. Additionally, the binding of apoptotic cells to macrophage receptor CD36 (cluster of differentiation 36) inhibits the production of pro-inflammatory cytokines such as TNF-*α*, IL-1*β *and IL-12; this binding also increases the secretion of TGF-*β *and IL-10 [13].

## Related work

This section presents and discusses other models found in the literature to model the innate HIS. Essentially, two distinct approaches are used: ordinary differential equations (ODEs) and partial differential equations (PDEs).

## Models based on ODEs

The authors of [14] presented a model of inflammation that is based on ODEs and considers three types of cells/molecules: the pathogen and two inflammatory mediators. This model was able to reproduce some experimental results depending on the values used for initial conditions and parameters. The authors described the results of the sensitivity analysis, which suggests some therapeutic strategies. Their work was then extended [15] to investigate the influence of time on an anti-inflammatory response. The mathematical model presented in [15] consists of a system of ODEs with four equations that model: a) the pathogen; b) the active phagocytes; c) tissue damage; and d) anti-inflammatory mediators. The source term of the phagocytes, in other words, a term that models the entry of new phagocytes into the infected tissue, is a function that depends on a) the concentration of phagocytes; b) the concentration of pathogens; and c) tissue damage. This term models the different interactions that phagocytes can undergo during an immune response, whether the interactions are direct or mediated by cytokines. In the interaction mediated by cytokines, they consider only the implicit presence of cytokines. For example, in an immune response, the interaction of phagocytes with tissue is mediated by pro-inflammatory cytokines produced by infected epithelial tissue cells, and this relationship is modeled directly in the source term of the phagocytes. This representation contrasts with the model proposed in the current work, where cytokines and all their interactions are explicitly represented.

A new adaptation of the first model [14] was proposed to simulate many scenarios involving repeated doses of endotoxin [16]. This work applied results obtained through experiments using mice to guide *in silico *experiments seeking to reproduce these results qualitatively. The mathematical model represents the key aspects of an acute inflammatory response, specifically when repeated doses of endotoxin are administered. This model replaces the pathogen equation proposed in the authors' previous work [15] with an equation incorporating the endotoxin. In their simulations, they observed that the timing and magnitude of endotoxin doses, as well as the dynamics between pro- and anti-inflammatory mediators, are key to distinguishing between potentiation and tolerance phenomena [16]. The authors also argued that their model, although simplified, nevertheless incorporates sufficiently complex dynamics to qualitatively reproduce a set of experimental results associated with different endotoxin administrations in mice.

One final work [17] developed a more complete system of ODEs that models acute inflammation. This model includes macrophages, neutrophils, dendritic cells, TH1 cells, blood pressure, tissue trauma, effector elements such as iNOS, ${\text{NO}}_{\text{2}}^{-}$ and ${\text{NO}}_{3}^{-}$, pro-inflammatory and anti-inflammatory cytokines, and coagulation factors. In this model, as well as our own (described in detail in the next section), neutrophils and macrophages are directly activated by LPS. Moreover, activation also occurs indirectly by way of various stimuli consistently elicited after a trauma or hemorrhage. However, the model proposed by [17] does not explicitly include initial events of inflammation such as mast cell degranulation and complement activation, although these factors were incorporated implicitly into cytokine and endotoxin dynamics. The model also includes anti-inflammatory cytokines such as IL-10 and TGF *β*, in addition to soluble receptors for pro-inflammatory cytokines. The authors argued that their model proved useful in simulating the inflammatory response induced in mice by endotoxin, trauma and surgery or surgical bleeding, as it can predict levels of TNF, IL-10, IL-6 and reactive products of NO (${\text{NO}}_{\text{2}}^{-}$ and ${\text{NO}}_{3}^{-}$) to some extent.

## Models based on PDEs

The model proposed by Su *et al *[18] uses a system of PDEs to represent the spatial dynamics of the innate and adaptive immune systems. It considers the simplest form of antigens, the molecular constituents of pathogen patterns, taking into account all the basic factors of an immune response: antigens, cells of the immune system, cytokines and chemokines. This model captures the following stages of immune response: recognition, initiation, effector response and resolution of infection or change to a new steady state. Accordingly, it can reproduce important phenomena such as a) the temporal order of cell arrival at the site of infection; b) antigen presentation by dendritic cells, macrophages and the involvement of regulatory T cells (Treg) in the resolution of the immune response; c) the production of pro-inflammatory and anti-inflammatory cytokines; and d) chemotaxis. This model has formed the basis for the development of our work.

## Mathematical model

The complete modeling of the HIS demands that a huge amount of work be performed by a large multidisciplinary team. In this work, we focus on a specific task: the development of a mathematical model of the innate immune response to the injection of LPS in a section of tissue, as well as such a model's computational implementation. One motivation for developing a model of the innate immune system is the fact that few such models are available in the literature; the majority of available models solely focus on the adaptive immune system. Another reason in favor of modeling the innate immune system is that many diseases result from the malfunction of the innate immune system; for these diseases, our proposed model could contribute to the definition of therapeutic strategies. In addition, a better comprehension of the inner workings of the separate parts composing the innate immune system is fundamental to a better understanding of immune response as a whole, as the innate immune system is responsible for both initiating the immune response and triggering the adaptive immune system.

Our objective is to develop a parameterized mathematical model of the human innate immune system that simulates the immune response occurring in a generic tissue. To achieve this goal, we first build a model of the immune response to LPS. We have chosen to use LPS because it is the major component of the outer membrane of Gram-negative bacteria, acting as an endotoxin substance that elicits strong immune responses; thus, it represents a vast number of inflammatory diseases. However, our proposed model is generic in the sense that it can be easily adapted to specific pathogens and distinct types of tissue through the adjustment of its parameters.

The mathematical model simulates the temporal and spatial behavior of lipopolysaccharide (*LPS*), macrophages, neutrophils (*N*), apoptotic neutrophils (*ND*), pro-inflammatory cytokines (*CH*), anti-inflammatory cytokine (*AC*) and protein granules (*G*). Macrophages are present in two states of readiness: resting (*RM*) and *hyperactivated *(*AM*). The different subsets of protein granules [19] released by neutrophils during their extravasation from blood vessels to the tissues are represented by a unique variable. Additionally, we must stress that the equations modeling pro- and anti-inflammatory cytokines are generic in the sense that they model the role of distinct cytokines taking part in the inflammatory process. Equation parameters can be adjusted to model the role of a specific pro- or anti-inflammatory cytokine.

**Figure 1:**
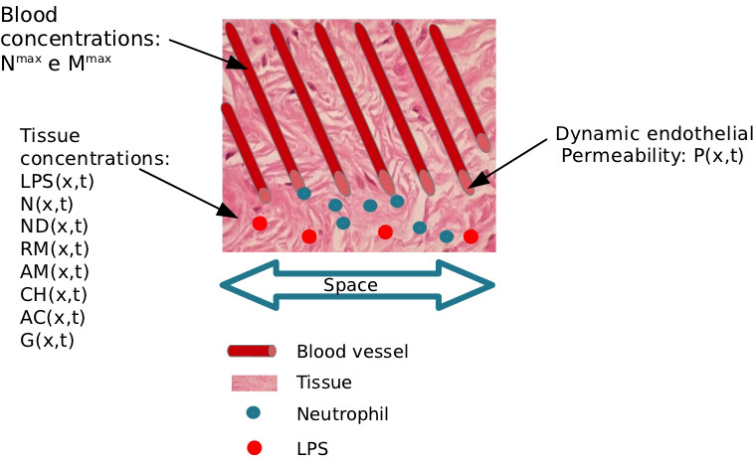
**Two-domain model**. Schematic representation of the two-domain model. The extravasation of neutrophils (or macrophages) from blood to tissue depends on local permeability, *P*(*x, t*), and on the difference between local concentrations of neutrophils in the two domains, *N^ max^*(*x, t*) - *N*(*x, t*). The permeability of the endothelium, which separates the two domains, varies with regard to time and space and depends on the local presence of pro-inflammatory cytokines and protein granules.

Our model extends the model proposed by Su *et al *[18] by considering a macroscopic or homogenized view of a tissue. In [18], the exchange between the vascular system (arterioles and vessels) and tissue was assumed to occur only at the boundaries of the domain, via Dirichlet boundary conditions. Our model allows each point of the tissue to be irrigated by arterioles and vessels, so that cells in the blood stream can enter into the tissue at any point. This is equivalent to a two-domain model, in which one domain represents the concentration of immune cells in the vascular system (in our case, neutrophils, *N ^max^*(*x, t*), and macrophages, *M^max^*(*x, t*)) and the other domain represents the different cells and molecules present in the tissue (our model considers lipopolysaccharide (*LPS*), neutrophils (*N*), apoptotic neutrophils (*ND*), pro-inflammatory cytokines (*CH*), anti-inflammatory cytokines (*AC*), protein granules (*G*), resting (*RM*) and *hyperactivated *(*AM*) macrophages). Communication between the two different domains is possible and is modeled by permeabilities that vary in space and time and may depend on the concentration of different cells and molecules (in our model, the endothelium permeability of neutrophils depends on the concentration of *CH*, whereas the permeability to macrophages depends on the concentration of both *CH *and *G*). Figure 1 presents our two-domain macroscopic model.

The main characteristics of the proposed model are:

• Macrophages and neutrophils cooperate to mount a more effective and intense response against the LPS;

• The endothelium's permeability may vary with time and space and also depends on the local concentration of pro-inflammatory cytokine and protein granules, as depicted by Figure 1;

• Active macrophages regulate immune responses through the production of anti-inflammatory cytokines and the phagocytosis of apoptotic neutrophils;

• Anti-inflammatory cytokines perform a key role in the control of the inflammatory response, avoiding a state of persistent inflammation after the complete elimination of LPS.

**Figure 2:**
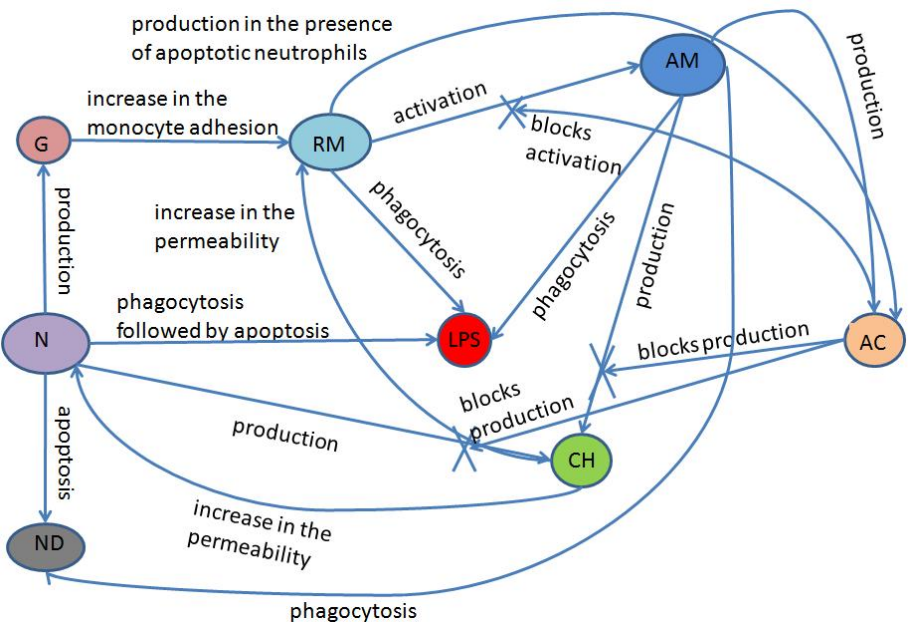
**Model's relations**. Relations among the components of the model.

Figure 2 depicts the relationships among all of the model's components. Neutrophils, resting macrophages and active macrophages phagocytose the LPS. The neutrophils then undergo apoptosis, which may or may not be induced by the phagocytosis process. In this different state, apoptotic neutrophils cannot perform phagocytosis or produce pro-inflammatory cytokines; as a result, apoptotic neutrophils are eliminated from the body after being phagocytosed by active macrophages. The number of apoptotic neutrophils in the serum is an indirect indication of the probability that the immune response will cause tissue damage, because apoptotic neutrophils will die after a period of time, releasing cytotoxic granules and degradation enzymes in the medium that can cause tissue damage. Neutrophils produce pro-inflammatory cytokines, such as TNF-*α *and IL-8, as well as protein granules, which allow the direct activation and adhesion of monocytes in the endothelium of blood vessels, facilitating monocytes' extravasation into the tissue. The resting macrophages become active when they recognize the LPS. The pro-inflammatory cytokines produced by neutrophils and active macrophages increase the permeability of the blood vessels; consequently, more neutrophils and monocytes are recruited to the tissue. In addition, the pro-inflammatory cytokines act as a chemoattractant substance to the resting macrophages, active macrophages and neutrophils. The production of the pro-inflammatory cytokine is blocked when an active macrophage or neutrophil comes in contact with an anti-inflammatory cytokine. Macrophage activation is also blocked by the action of an anti-inflammatory cytokine, which is produced by active macrophages and by resting macrophages that are in contact with apoptotic neutrophils.

Below, we provide the equations derived from the model. Equation 1 provides the LPS differential equation.

(1) $$\left\{\begin{array}{c} RM_{activation}=\frac{\phi_{RM|LPS}.RM.LPS}{\left(1+\theta_{AC}.AC\right)}\\ \frac{\partial LPS}{\partial t}=-\mu_{LPS}LPS-RM_{activation}-\left({\lambda_{N|}}_{LPS}N+{\lambda_{AM}{}_{|}}_{LPS}AM\right).LPS+D_{LPS}\text{Δ}LPS\\ LPS\left(x,0\right)=LPS_{0},\frac{\partial LPS\left(.,t\right)}{\partial n}{|}_{\partial \text{Ω}}=0 \end{array}\right)$$

**Figure 3:**
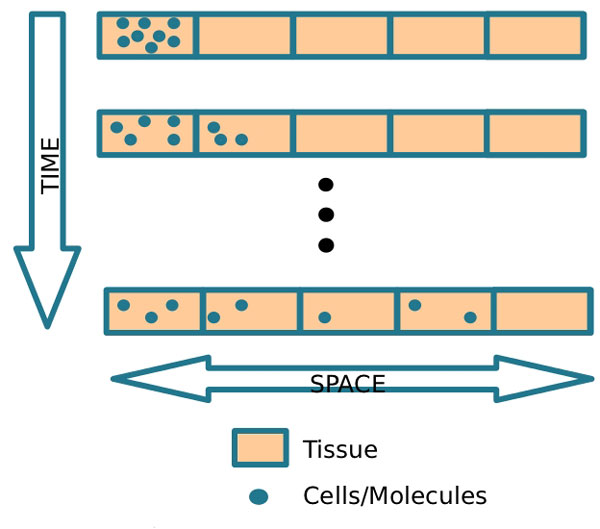
**Diffusion process**. Schematic representation of the diffusion process.

In this equation, *μ_LPS_LPS *denotes the decay of LPS, where *μ_LPS _*is the rate of decay, *RM_activation _*denotes the activation of resting macrophages, where *ϕ*_*RM*|*LPS *_is the rate of activation. This activation occurs when resting macrophages recognize the LPS, after which macrophages phagocytose the LPS. *λ*_*N*|*LPS*_.*N *denotes the phagocytosis of LPS by neutrophils, where *λ*_*N*|*LPS *_is the rate of this phagocytosis. *λ_AM|LPS_*.*AM *denotes the phagocytosis of LPS by active macrophages, where *λ_AM|LPS _*is the rate of this phagocytosis. *D_LPS_*Δ*LPS *denotes LPS diffusion, whereas *D_LPS _*represents the diffusion coefficient. Figure 3 presents a schematic representation of the diffusion process implemented by the diffusion operator, *D_LPS_*Δ*LPS*, and illustrates the diffusion of cells through the tissue. Diffusion is defined as the spread of particles from regions of higher concentration to regions of lower concentration.

The differential equation corresponding to the resting macrophage (RM) is given in Equation 2.

(2) $$\left\{\begin{array}{c} RM_{P}=\left(P_{RM}^{max}-P_{RM}^{min}\right).\frac{CH}{\left(CH+keqch\right)}+P_{RM}^{min}\\ RM_{Q}=\left(Q_{RM}^{max}-Q_{RM}^{min}\right).\frac{G}{\left(G+keq\_g\right)}+Q_{RM}^{min}\\ source_{RM}=\left(RM_{P}+RM_{Q}\right).\left(M^{max}-\left(RM+AM\right)\right)\\ \frac{\partial RM}{\partial t}=-\mu_{RM}RM-RM_{activation}+D_{RM}\text{Δ}RM+source_{RM}-\nabla .\left(\chi_{RM}RM\nabla CH\right)\\ RM\left(x,0\right)=RM_{0},\frac{\partial RM\left(.,t\right)}{\partial n}{|}_{\partial \text{Ω}}=0 \end{array}\right)$$

**Figure 4:**
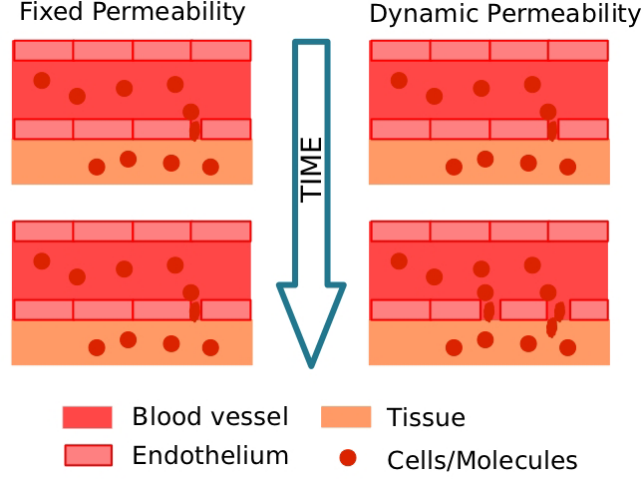
**Permeability**. Representation of the differences between fixed and dynamic permeabilities.

*RM_P _*and *RM_Q _*denote the increase in endothelium permeability and its effects on monocyte extravasation. The permeability of blood vessel endothelium is modeled by a Hill equation [20], which also has been used to model drug dose-response relationships [21]. The idea is to model the increase in the permeability of the endothelium in accordance with the number of pro-inflammatory cytokines deposited on the endothelium. Figure 4 illustrates the effect of increasing blood vessel permeability. We can see that the space between two neighboring endothelial cells increases, allowing more cells to extravasate to the tissue. The dynamic permeability depends on the cytokine concentration.

The calculation of *RM_P _*involves the following parameters: a) $P_{RM}^{max}$, the maximum endothelium permeability induced by the pro-inflammatory cytokine; b) $P_{RM}^{min}$, the minimum endothelium permeability induced by the pro-inflammatory cytokine; and c) *keqch*, the number of pro-inflammatory cytokines that exert 50% of the maximum effect on permeability.

*RM_Q _*denotes the increase in endothelium permeability induced by protein granules, and its calculation is similar to that of *RM_P_*, except for the parameters involved: $Q_{RM}^{max}$, $Q_{RM}^{min}$ and *keq_g. source_RM _*denotes the source term of macrophages, which is related to the number of monocytes that will enter into the tissue from the blood vessels. This number depends on the endothelium permeability *RM_P _*+ *RM_Q _*and on the number of monocytes appearing in the blood (*M^max^*).

*μ_RM _RM *denotes resting macrophage apoptosis, where *μ_RM _*is the apoptosis rate. *RM_activation_*, as explained above, models the activation of resting macrophages and denotes the number of resting macrophages that are becoming active. The term *D_RM_*Δ*RM *denotes the resting macrophage diffusion, where *D_RM _*is the diffusion coefficient. ∇.(*χ_RM_RM*∇*CH*) denotes the resting macrophage chemotaxis, where *χ_RM _*is the chemotaxis rate.

**Figure 5:**
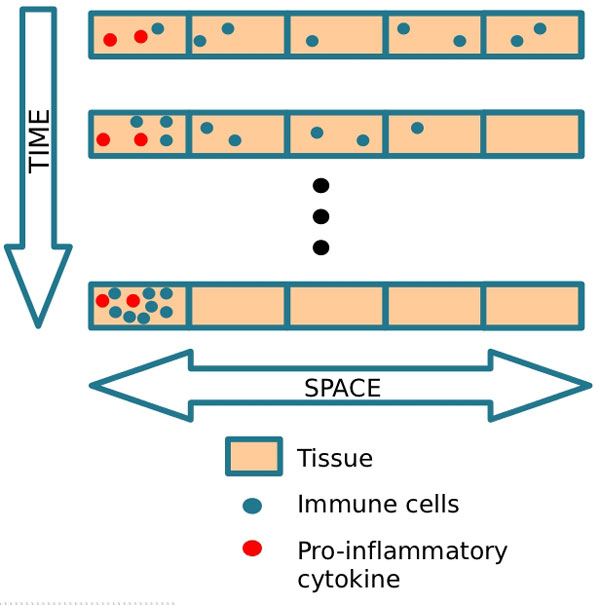
**Chemotaxis process**. Schematic representation of chemotaxis.

Figure 5 provides a schematic representation of the chemotaxis process implemented by the chemotaxis operator, ∇.(*χ_RM_RM*∇*CH*). Chemotaxis is the phenomenon by which cells direct their own movements according to certain chemicals present in their environment.

The differential equation corresponding to the active macrophage (AM) is given in Equation 3.

(3) $$\left\{\begin{array}{c} \frac{\partial AM}{\partial t}=-\mu_{AM}AM+RM_{activation}+D_{AM}\text{Δ}AM-\nabla .\left(\chi_{AM}AM\nabla CH\right)\\ AM\left(x,0\right)=AM_{0},\frac{\partial AM\left(.,t\right)}{\partial n}{|}_{\partial \text{Ω}}=0 \end{array}\right)$$

Above, *μ_AM_AM, D_AM_*Δ*AM*, and ∇.(*χ_AM_AM*∇*CH*) denote the active macrophage apoptosis, diffusion, and chemotaxis, respectively, whereas *μ_AM_, D_AM_*, and *χ_AM _*are the apoptosis rate, diffusion coefficient, and chemotaxis rate, respectively.

The differential equation for the pro-inflammatory cytokine (CH) is given in Equation 4.

(4) $$\left\{\begin{array}{c} \frac{\partial CH}{\partial t}=-\mu_{CH}CH+\left(\left(\beta_{CH|N}.N.LPS+\beta_{CH|AM}.AM.LPS\right).\left(1-\frac{CH}{chInf}\right)\right)/\left(1+\theta_{AC}.AC\right)+\\ +D_{CH}\text{Δ}CH\\ CH\left(x,0\right)=CH_{0},\frac{\partial CH\left(.,t\right)}{\partial n}{|}_{\partial \text{Ω}}=0 \end{array}\right)$$

In this equation, *μ_CH_CH *denotes the pro-inflammatory cytokine decay, where *μ_CH _*is the decay rate. *β*_*CH*|*N*_.*N *denotes the pro-inflammatory cytokine production by the neutrophils, where *β*_*CH*|*N *_is the production rate. *β*_*CH*|*AM*_.*AM *denotes the pro-inflammatory cytokine production by active macrophages, where *β_CH|AM _*is the production rate. The saturation of cytokine production by active macrophages is calculated by the equation $\left(1-\frac{CH}{chInf}\right)$, where *chInf * is an estimate of the maximum quantity of pro-inflammatory cytokine supported by the tissue. The production of pro-inflammatory cytokine decreases when anti-inflammatory cytokine acts on the producing cells. This influence of anti-inflammatory cytokine is denoted by the expression 1/(1 + *θ_AC_.AC*). *D_CH_*Δ*CH *models pro-inflammatory cytokine diffusion, where *D_CH _*is the diffusion coefficient.

The neutrophil differential equation (N) is given in Equation 5.

(5) $$\left\{\begin{array}{c} P_{N}=\left(P_{N}^{max}-P_{N}^{min}\right).\frac{CH}{CH+Keqch}+P_{N}^{min}\\ source_{N}=P_{N}.\left(N^{max}-N\right)\\ \frac{\partial N}{\partial t}=-\mu_{N}N-\lambda_{LPS|N}LPS.N+D_{N}\text{Δ}N+source_{N}-\nabla .\left(\chi_{N}N\nabla CH\right)\\ N\left(x,0\right)=N_{0},\frac{\partial N\left(.,t\right)}{\partial n}{|}_{\partial \text{Ω}}=0 \end{array}\right)$$

In this equation, *P_N _*denotes the increase in endothelium permeability and its effects on neutrophil extravasation. In the top equation, $P_{N}^{max}$ is the maximum endothelium permeability induced by pro-inflammatory cytokines, $P_{N}^{min}$ is the minimum endothelium permeability induced by pro-inflammatory cytokines and *keqch *is the number of pro-inflammatory cytokines that exert 50% of the maximum effect on endothelium permeability.

Here, *μ_N_N *denotes neutrophil apoptosis, where *μ_N _*is the rate of apoptosis. *λ_LPS|N _LPS.N *denotes the neutrophil apoptosis induced by phagocytosis, where *λ_LPS|N _*represents the rate of this induced apoptosis. The term *D_N_*Δ*N *denotes neutrophil diffusion, where *D_N _*is the diffusion coefficient. *source_N _*represents the source term of neutrophil, i.e., the number of neutrophils entering the tissue from the blood vessels. This number depends on the endothelium permeability (*P_N_*) and on the number of neutrophils in the blood (*N^max^*). The term ∇.(*χ_N_N*∇*CH*) denotes the chemotaxis process of the neutrophils, where *χ_N _*represents the chemotaxis rate.

The differential equation corresponding to the apoptotic neutrophil (ND) is given in Equation 6.

(6) $$\left\{\begin{array}{c} \frac{\partial ND}{\partial t}=\mu_{N}N+\lambda_{LPS|N}LPS.N-\lambda_{ND|AM}ND.AM+D_{ND}\text{Δ}ND\\ ND\left(x,0\right)=ND_{0},\frac{\partial ND\left(.,t\right)}{\partial n}{|}_{\partial \text{Ω}}=0 \end{array}\right)$$

Here, note that *μ_N_N *and *λ_LPS|N_LPS.N *were defined previously, whereas *λ_ND|AM_ND.AM *denotes the phagocytosis of the apoptotic neutrophil carried out by active macrophages, and *λ_ND|AM _*is the rate of this phagocytosis. *D_ND_*Δ*ND *models the apoptotic neutrophil diffusion, where *D_ND _*is the diffusion coefficient.

The differential equation for protein granules (G) is given in Equation 7.

(7) $$\left\{\begin{array}{c} \frac{\partial G}{\partial t}=-\mu_{G}G+\alpha_{G|N}.source_{N}.\left(1-\frac{G}{gInf}\right)+D_{G}\text{Δ}G\\ G\left(x,0\right)=G_{0},\frac{\partial G\left(.,t\right)}{\partial n}{|}_{\partial \text{Ω}}=0 \end{array}\right)$$

*μ_G_G *models the decay of the granules, where *μ_G _*is the decay rate. *α_G|N_*.*source_N _*denotes the production of protein granules by neutrophils extravasating from the blood into inflamed tissue, where *α_G|N _*is a dimensionless constant. The saturation of protein granule production is calculated by the expression $\left(1-\frac{G}{gInf}\right)$, where *gInf * is the maximum number of protein granules. *D_G_*Δ*G *models protein granule diffusion, where *D_G _*is the diffusion coefficient.

The differential equation for the anti-inflammatory cytokine (AC) is given in Equation 8.

(8) $$\left\{\begin{array}{c} \frac{\partial AC}{\partial t}=-\mu_{AC}AC+\left(\beta_{RM|ND}.RM.ND+\alpha_{AC|AM}.AM\right).\left(1-\frac{AC}{acInf}\right)+D_{AC}\text{Δ}AC\\ AC\left(x,0\right)=AC_{0},\frac{\partial AC\left(.,t\right)}{\partial n}{|}_{\partial \text{Ω}}=0 \end{array}\right)$$

In this equation, *μ_AC_AC *denotes the anti-inflammatory cytokine decay, where *μ_AC _*represents the decay rate. *β_RM|ND_.RM.ND *denotes the anti-inflammatory cytokine production by the resting macrophages in the presence of apoptotic neutrophils, where *β_RM|ND _*is the rate of this production. *α_AC|AM_.AM *denotes the anti-inflammatory cytokine production by active macrophages, where this production has rate *α_AC|AM _*and saturation $\left(1-\frac{AC}{acInf}\right)$, where *acInf * is the maximum number of anti-inflammatory cytokines in the tissue. *D_AC_*Δ*AC *models the anti-inflammatory cytokine diffusion, where *D_AC _*is the diffusion coefficient.

## Implementation

The numerical method that we have applied to our mathematical model is presented in our previous work [2].

We executed some convergence tests to test the implementation of our numerical method. In short, we assumed that the correct solution derived from the results of a very refined mesh, where the refinement was in terms of time (*dt *= 10^-6^*day*) and space (*deltaX *= 0.1*mm*). To show convergence with respect to time, we selected two new values for *dt, dt*1 = 4.0 × 10^-6^*day *and *dt*2 = 8.0 × 10^-6^*day*. We applied the L2-norm to compute the errors when using *dt*1 and *dt*2 for our refined mesh. We observed that the error when using *dt*2 was 2.3 times greater than the error obtained with *dt*1. Therefore, as theoretically predicted, our numerical scheme is first-order accurate with respect to time. We then conducted the same analysis for convergence with respect to space, choosing two new values of *deltaX, dx*1 = 0.4*mm *and *dx*2 = 0.8*mm*. The L2-norm error when using *dx*2 was 2.03 times greater than the error obtained with *dx*1. Once again, the values obtained were as expected, as we were using a first-order discretization (upwind) in space. These results gave us confidence that our numerical solver had been correctly implemented.

## Numerical experiments

The model's initial conditions and parameters are given in Tables 1 and 2, respectively. In our simulations, we assumed a one-dimensional domain of 5 *mm *length and a simulation time of 5 days. In fact, this one-dimensional model is a simplification of a 3D block model in that we have assumed that the lengths associated with *y *and *z *are much smaller than the length associated with *x*.

**Table 1:** Initial Conditions

Parameter	Value	Unit
*LPS*_0_	100: 0<*x *< 1	10^4^*cells/mm*^3^
*LPS*_0_	0: 1 ≤ *x *< 5	10^4^*cells/mm*^3^
*RM*_0_	1: 0 <*x *< 5	10^4^*cells/mm*^3^
*AM*_0_	0: 0 <*x *< 5	10^4^*cells/mm*^3^
*CH*_0_	0: 0 <*x *< 5	10^4^*cells/mm*^3^
*N*_0_	0: 0 <*x *< 5	10^4^*cells/mm*^3^
*ND*_0_	0: 0 <*x *< 5	10^4^*cells/mm*^3^
*G*_0_	0: 0 <*x *< 5	10^4^*cells/mm*^3^
*AC*_0_	0: 0 <*x *< 5	10^4^*cells/mm*^3^

**Table 2:** Parameters

Parameter	Value	Unit	Reference
*ϕ_RM|LPS_*	0.1	1/(*cells/mm*^3^).day	[18]**
*θ_AC _*	1	1/(*cells/mm*^3^)	estimated*
*μ_LPS _*	0.005	1/day	[18]
*λ_N|LPS _*	0.55	1/(*cells/mm*^3^).day	[18]
*λ_AM|LPS _*	0.8	1/(*cells/mm*^3^).day	[18]
*D_LPS _*	2000	*μm*^2^/day	estimated*
*$P_{RM}^{max}$*	0.1	1/day	estimated*
*$P_{RM}^{min}$*	0.01	1/day	estimated*
*$Q_{RM}^{max}$*	0.5	1/day	estimated*
*$Q_{RM}^{min}$*	0	1/day	estimated*
*keqch *	1	*cells/mm*^3^	estimated*
*keq_g*	1	*cells/mm*^3^	estimated*
*M^max ^*	6	*cells/mm*^3^	estimated*
*μ_RM _*	0.033	1/day	[18]
*D*_RM_	4320	*μm*^2^/day	[29,30]
*μ_RM _*	3600	*μm*^2^/day	[31-33]
*μ_AM _*	0.07	1/day	[18]
*D*_AM_	3000	*μm*^2^/day	[29,30]
*μ_AM _*	4320	*μm*^2^/day	[31-33]
*μ_CH _*	7	1/day	[18]**
*β_CH|N _*	1	1/(*cells/mm*^3^).day	[34]
*β_CH|AM _*	0.8	1/(*cells/mm*^3^).day	[34]
*chInf *	3.6	*cells/mm*^3^	[8]**
*D*_CH_	9216	*μm*^2^/day	[18,29]
*$P_{N}^{max}$*	11.4	1/day	[35]**
*$P_{N}^{min}$*	0.0001	1/day	estimated*
*keqch *	1	*cells/mm*^3^	estimated*
*N^max ^*	8	*cells/mm*^3^	estimated*
*μ_N _*	3.43	1/day	[36]
*λ_LPS|N _*	0.55	1/(*cells/mm*^3^).day	[18]
*D_N _*	12096	*μm*^2^/day	[37]
*μ_N _*	14400	*μm*^2^/day	[38]
*λ_ND|AM _*	2.6	1/(*cells/mm*^3^).day	[18]
*D_ND _*	0.144	*μm*^2^/day	[18]**
*μ_G _*	5	1/day	estimated*
*α_G|N _*	0.6	dimensionless	estimated*
*gInf *	3.1	*cells/mm*^3^	estimated*
*D_G _*	9216	*μm*^2^/day	estimated*
*μ_AC _*	4	1/day	estimated*
*β_RM|ND _*	1.5	1/(*cells/mm*^3^).day	estimated*
*α_AC|AM _*	1.5	dimensionless	estimated*
*acInf *	3.6	*cells/mm*^3^	[8]**
*D_AC _*	9216	*μm*^2^/day	[29]

In this paper, we obtained parameter values for humans whenever they were available. We chose values for the initial concentrations of LPS according to the work of the authors in [22]. In their experiments, *E. coli *cells were inoculated intradermally (10^8^) into normal and neutropenic rabbits. They reported that all bacteria and inflammatory cells were contained in this 1.5 cm diameter biopsy and restricted to its 0.2 cm thick layer of dermal collagen. Thus, the volume of dermis in which the *E. coli *cells were contained was approximately 0.35 cm^3 ^[23]. This finding suggested us a value of *LPS*_0 _= 100.0 × 10^4 ^cells/*mm*^3 ^.

In Table 2, parameters marked with * were adjusted to qualitatively reproduce the results obtained in several studies of the immune response to LPS. In the case of LPS, we adjust the equation parameters to obtain an exponential decrease, as shown in [24]. The results of the concentration of pro-inflammatory cytokines over time are qualitatively similar to those obtained in some experimental works [25-27]. The time course for the anti-inflammatory cytokine is qualitatively similar to the results in [25]. An important feature present in our model is the inhibition of the production of pro-inflammatory cytokines by neutrophils through the action of anti-inflammatory cytokines [10]. The protein granule model behavior is based on existing work [28]. The parameters marked with ** were based on the values given in the references but were adjusted due to the use of distinct units (for example, from L to *mm*^3^) or to fit in a 5 *mm *tissue.

**Figure 6:**
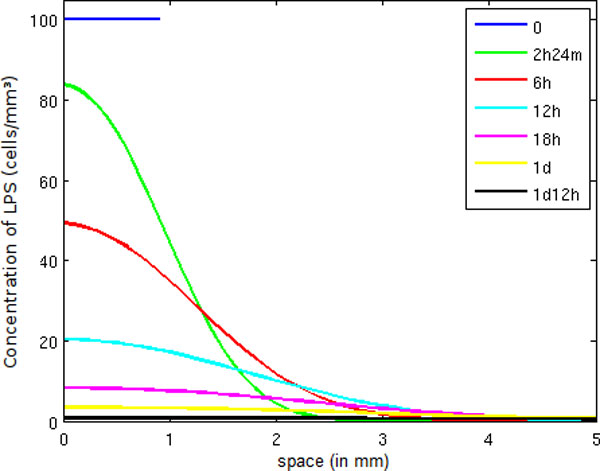
**LPS concentration in space**. Temporal evolution of the spatial distribution of LPS.

In Figure 6, we initially inject LPS only into a small part of the tissue. As time progresses, we can see two important phenomena occurring: the diffusion of LPS through the tissue and the decrease of LPS mainly due to the action of neutrophils and macrophages.

**Figure 7:**
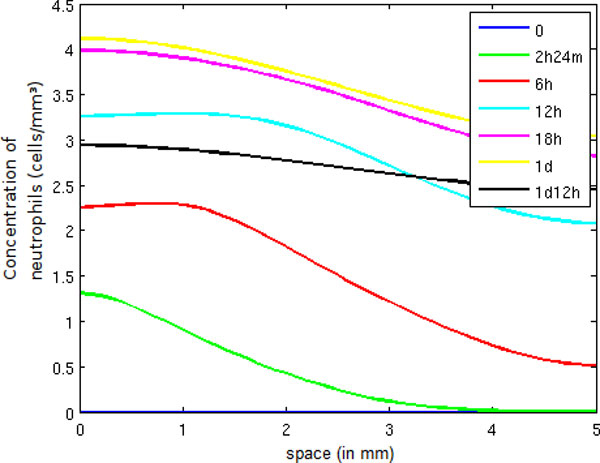
**Neutrophil concentration in space**. Temporal evolution of the spatial distribution of neutrophil.

In the case of neutrophils (Figure 7), we can witness an increase in neutrophil population mainly in regions of tissue having higher levels of LPS. This increase happens because of an increase in endothelium permeability in addition to the chemotaxis process attracting neutrophils to regions possessing more pro-inflammatory cytokines. When the amount of LPS is low, the neutrophil population stops growing and starts to decrease because fewer neutrophils are entering into the tissue.

**Figure 8:**
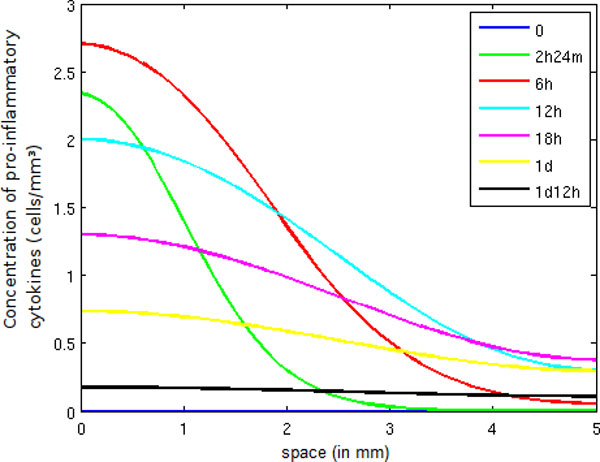
**Pro-inflammatory cytokine concentration in space**. Temporal evolution of the spatial distribution of pro-inflammatory cytokine.

**Figure 9:**
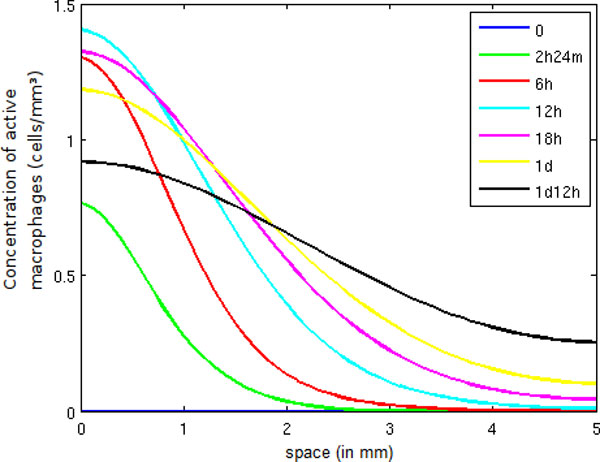
**Active macrophage concentration in space**. Temporal evolution of the spatial distribution of active macrophage.

In Figure 8, we observe an increase of pro-inflammatory cytokines until 6 hours, when a large number of neutrophils are present in the tissue. Afterwards, the number of pro-inflammatory cytokines decreases, mainly due to the presence of a large number of active macrophages. Consequently, the anti-inflammatory cytokine population increases. The decrease of *CH *has many important consequences: fewer neutrophils and monocytes are migrating to the inflamed tissue, and fewer macrophages are becoming active, as can be observed in Figure 9. This figure shows that the active macrophage population grows until 12 hours and then starts to decrease because, as explained before, anti-inflammatory cytokines inhibit the activation of resting macrophages.

## Comparison of different scenarios

To show the importance of some cells, molecules and processes in the dynamics of the innate immune response, we performed a set of simulations under different scenarios. Each simulation begins with a simple scenario in which we assume that only macrophages participate in the immune response to LPS (Case 1). We then consider progressively more complex scenarios. In each subsequent scenario, a new set of equations and terms are added to the previous one until the complete scenario is obtained (Case 5).

A description of each case is given below:

• Case 1: only macrophages participate in the immune response. Resting tissue-resident macrophages are responsible for the initial response to LPS.

• Case 2: considers a) the production of pro-inflammatory cytokines by active macrophages; and b) all effects of pro-inflammatory cytokines, such as the increase in permeability and chemotaxis.

• Case 3: incorporates neutrophils into the model, which participate in the immune response as a major phagocytic leukocyte. They are also responsible for producing pro-inflammatory cytokines.

• Case 4: incorporates protein granules into the model, which are produced by neutrophils and contribute to an increase in the endothelium's permeability, allowing more monocytes to enter into the tissues and differentiate in resting macrophages.

• Case 5: incorporates anti-inflammatory cytokines into the model. In this case, anti-inflammatory cytokines block the production of pro-inflammatory cytokines by the neutrophils and active macrophages. In addition, anti-inflammatory cytokines block the activation of resting macrophages.

**Figure 10:**
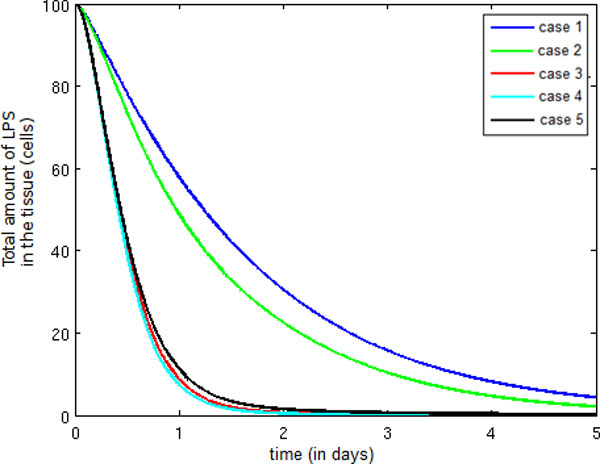
**Temporal evolution LPS**. Temporal evolution of the total quantity of LPS.

Figure 10 depicts the temporal evolution of the total amount of LPS in the tissue. Observe that the introduction of pro-inflammatory cytokines in Case 2 causes a small decrease in the amount of LPS when compared to Case 1. This decrease has occurred because our model considers the pro-inflammatory cytokine influence on monocyte migration to be almost negligible.

In Case 3, the decrease in LPS has been accelerated due to the presence of neutrophils migrating into the tissue in huge quantities. The number of neutrophils in the tissue is enough to control the infection.

In Case 4, observe that the extravasation of a second wave of monocytes (a consequence of the presence of protein granules produced by the neutrophils) has no impact on the potentiation of the immune response because the LPS has been almost completely eliminated. Note that the LPS decrease is smaller in case 5 than in cases 3 and 4. This fact is a consequence of the presence of anti-inflammatory cytokines in the model, which causes a decrease in the number of neutrophils and monocytes extravasating to the tissue.

**Figure 11:**
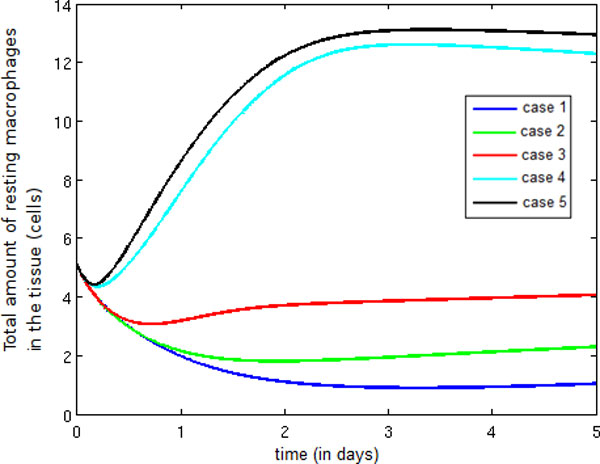
**Temporal evolution resting macrophage**. Temporal evolution of the resting macrophage population.

Figure 11 depicts the temporal evolution of the population of resting macrophages and demonstrates that the introduction of pro-inflammatory cytokines, neutrophils and protein granules (Cases 2, 3 and 4) contributes to an increase in endothelium permeability, which in turn allows the entry of more monocytes. As a consequence, the number of resting macrophages increases, an increase compounded between Cases 4 and 5 because anti-inflammatory cytokines are blocking the activation of resting macrophages.

**Figure 12:**
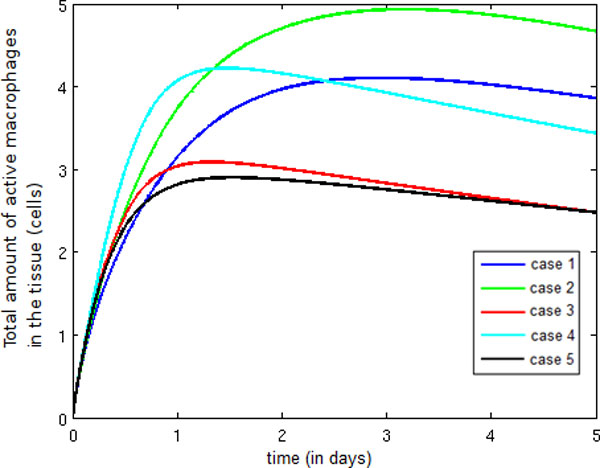
**Temporal evolution active macrophage**. Temporal evolution of the active macrophage population.

Figure 12 presents the temporal evolution of the active macrophage population. Observe that an increase in this population occurs from Case 1 to Case 2, which is due to the production of pro-inflammatory cytokines by active macrophages, which have also increased permeability and chemotaxis. In Case 3, the introduction of neutrophils contributes to a faster elimination of LPS, and as a result, less LPS is available to activate resting macrophages. When protein granules are included in the model (Case 4), we can observe an increase in the quantity of active macrophages. This population increase has occurred because protein granules allow the direct activation and adhesion of monocytes in the endothelium of blood vessels, thus facilitating the monocytes' extravasation to the tissues. Finally, a significant reduction in the total amount of active macrophages occurs in Case 5 due to the action of anti-inflammatory cytokines, which block the activation of resting macrophages. In addition, anti-inflammatory cytokines block the production of pro-inflammatory cytokines, causing a decrease in endothelium permeability and consequently in the number of monocytes extravasating to the tissue.

**Figure 13:**
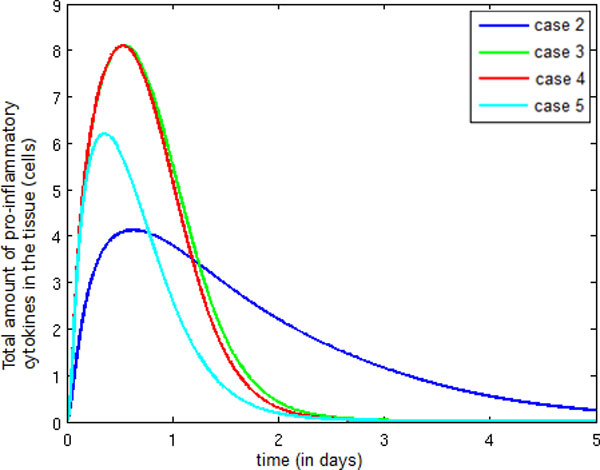
**Temporal evolution pro-inflammatory cytokine**. Temporal evolution of the pro-inflammatory cytokine population.

Observe the significant increase in the number of pro-inflammatory cytokines in Figure 13 between Cases 2 and 3. This increase is a direct consequence of the incorporation of neutrophils into the model, as neutrophils produce a huge amount of pro-inflammatory cytokines. No change occurs between Cases 3 and 4 because the entry of more monocytes into the tissue occurs during termination of the immune response, when the LPS available to activate the monocytes is small. In Case 5, the reduction in production of pro-inflammatory cytokines due to the action of anti-inflammatory cytokines is responsible for the decrease in the total quantity of pro-inflammatory cytokines.

**Figure 14:**
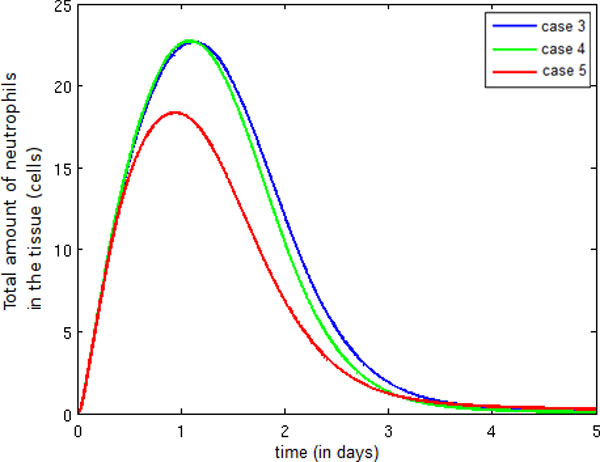
**Temporal evolution neutrophil**. Temporal evolution of the neutrophil population.

Figure 14 depicts the temporal evolution of the neutrophil population, whose increase is similar in Cases 3 and 4 due to the fact previously stated: the entry of more monocytes into the tissue occurs during termination of the immune response. In Case 5, the number of neutrophils into the infected tissue is smaller than in Case 4 because fewer pro-inflammatory cytokines are present in the tissue, which results in a reduction in the number of neutrophils migrating into the infected tissue.

**Figure 15:**
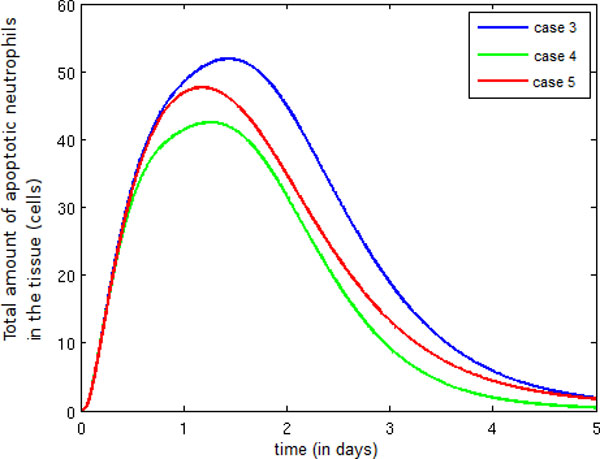
**Temporal evolution apoptotic neutrophil**. Temporal evolution of the apoptotic neutrophil population.

Figure 15 illustrates a small decrease in the number of apoptotic neutrophils between Cases 3 and 4. This reduction is a consequence of the presence of more active macrophages in Case 4 than in Case 3. In Case 5, the presence of fewer active macrophages in the tissue leads to a reduction in the number of apoptotic neutrophils that are phagocytosed.

**Figure 16:**
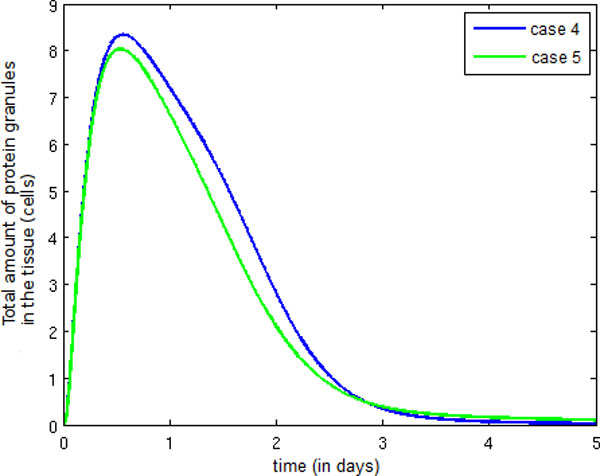
**Temporal evolution protein granule**. Temporal evolution of the protein granule population.

Figure 16 shows an increase in the number of protein granules between Case 4 and Case 5. In Case 4, the number of neutrophils migrating to the infected tissue is larger, causing an increase in protein granule production as well.

**Figure 17:**
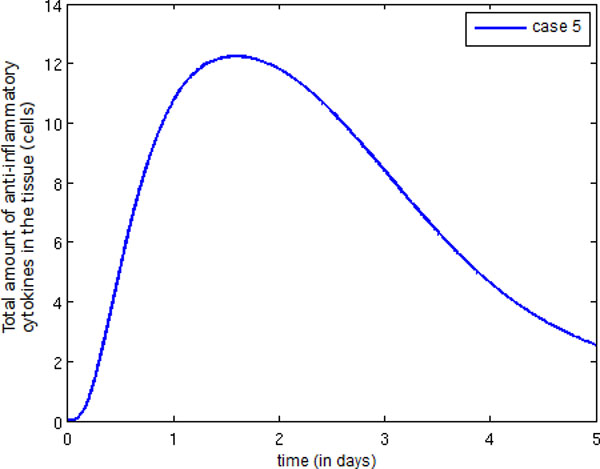
**Temporal evolution anti-inflammatory cytokine**. Temporal evolution of the anti-inflammatory cytokine population.

Finally, Figure 17 depicts the temporal evolution of the anti-inflammatory cytokine population. Observe that the number of anti-inflammatory cytokines increases after the termination of the infection (as shown in Figure 10).

## Conclusions and future works

In this work, we have presented a computational model for the dynamics of representative types of cells and molecules of the HIS during an innate response to the injection of LPS into a small section of tissue. To achieve this objective, we have proposed a mathematical model that incorporates the main interactions occurring between LPS and some cells and molecules of the innate immune system. The model proposes a macroscopic or homogenized view of tissue composed of two different domains: one domain represents the concentration of immune cells in the vascular system (in our case, neutrophils, *N_^max^_*(*x, t*), and macrophages, *M_^max^_*(*x, t*)), whereas the other domain represents the different cells and molecules present in the tissue (our model considers lipopolysaccharide (*LPS*), neutrophils (*N*), apoptotic neutrophils (*ND*), pro-inflammatory cytokines (*CH*), anti-inflammatory cytokines (*AC*), proteins granules (*G*), resting (*RM*) and *hyperactivated *(*AM*) macrophages). Communication between the two different domains is possible and is modeled by an endothelium permeability that varies in space and time and may depend also on the concentration of different cells and molecules (in our model, the endothelium's permeability to neutrophils depends on the concentration of *CH*, whereas its permeability to macrophages depends on *CH *and *G*).

The model proposed in this work has been able to reproduce several features present in immune responses, such as:

• the order of arrival of cells at the site of infection, as shown in [39];

• the coordination of macrophages and neutrophils to mount a more effective and intense response to LPS;

• the endothelium's dynamic permeability, which may depend on local concentrations of pro-inflammatory cytokines and protein granules;

• the important role of protein granules throughout the process of monocyte extravasation;

• the regulation of immune response by macrophages through the production of anti-inflammatory cytokines and the phagocytosis of apoptotic neutrophils;

• the crucial role of anti-inflammatory cytokines in the control of the inflammatory response, thus avoiding a state of persistent inflammation after the complete elimination of LPS.

In future work, we plan to implement a more complete mathematical model that includes new cells (such as natural killer and dendritic cells), molecules and other processes involved in the immune response. The model could be extended by any of the following methods: a) including the interaction between endothelial cells, LPS and some cytokines such as IL-1*β *and TNF-*α *[40]; b) incorporating the fact that high amounts of LPS also induce an increase in endothelium permeability [40]; c) considering the process of macrophage desensitization, in which high levels of LPS inhibit the production of TNF-*α *by macrophages [41]; d) taking into account that the TNF-*α *produced by macrophages induces the production of even more TNF-*α *[1]; and e) considering that the TNF-*α *has proapoptotic and antiapoptotic effects on macrophages and neutrophils. In low concentrations, TNF-*α *delays the apoptosis of macrophages and neutrophils and induces the production of pro-inflammatory cytokines, whereas in high concentrations, it induces apoptosis [41].

An important final step is the validation of our proposed model using experimental data. Of particular interest is the spatio-temporal modeling of microabscess formation, a very important research topic. For instance, [42-45] presents animal studies detailing the formation of liver abscess and microabscess by different types of infections. Epidermal microabscess formation by neutrophils was also evaluated in [46-48] and [22]. Infection of the heart by bacteria (bacterial myocarditis [49]) or by viruses (viral myocarditis [50]) is also correlated with microabscess formation by neutrophils. The interaction between tumor cells and inflammatory cells plays an important role in cancer initiation and progression and was investigated in [51] for the case of tumor-infiltrating neutrophils in pancreatic neoplasia, where the pattern of microabscess formation by neutrophils was reported once again. We acknowledge that this distinct pattern of formation can only be numerically reproduced and studied by models that capture the spatio-temporal dynamics of the HIS. Therefore, in the near future, we plan to extend our PDE model and adjust its parameters in the hopes of reproducing some of these experimental findings.

## Authors' contributions

RWS and ML have defined the methods and experiments. ABP has written the software code to implement the model and has performed all simulations. ABP and GCM have analyzed and interpreted the results. All authors have written the paper. They have read and approved the final manuscript.

## Competing interests

The authors declare that they have no competing interests.

## Declarations

This article has been published as part of *BMC Bioinformatics *Volume 14 Supplement 6, 2013: Selected articles from the 10th International Conference on Artificial Immune Systems (ICARIS). The full contents of the supplement are available online at http://www.biomedcentral.com/bmcbioinformatics/supplements/14/S4.
